# Sirt1 Inhibits Resistin Expression in Aortic Stenosis

**DOI:** 10.1371/journal.pone.0035110

**Published:** 2012-04-06

**Authors:** Sophie Carter, Stéphanie Miard, Catherine Roy-Bellavance, Louise Boivin, Zhuo Li, Philippe Pibarot, Patrick Mathieu, Frédéric Picard

**Affiliations:** Quebec Heart and Lung Research Center, Laval University, Québec, Québec City, Canada; Kaohsiung Chang Gung Memorial Hospital, Taiwan

## Abstract

The development of human calcified aortic stenosis (AS) includes age-dependent processes that have been involved in atherosclerosis, such as infiltration of macrophages in aortic valves, which then promote production of many pro-inflammatory cytokines, including resistin. However, the molecular mechanisms contributing to these processes are not established. Since Sirt1 has been shown to modulate macrophage biology and inflammation, we examined its levels in human AS and tested its impact on resistin expression. Sirt1 mRNA (p = 0.01) and protein (p<0.05) levels were reduced in explanted valves from AS patients (n = 51) compared to those from control (n = 11) patients. Sirt1 mRNA levels were negatively associated with resistin mRNA levels quantified in AS valves (p = 0.02). Stimulation of Sirt1 by resveratrol or virus-driven overexpression robustly diminished resistin mRNA and protein expression in macrophages, whereas down-regulation of Sirt1 triggered a large increase in resistin expression. These effects were direct, as chromatin immunoprecipitation assays showed that Sirt1 physically interacted with the resistin promoter region at an AP-1 response element. Moreover, Sirt1 blocked c-jun-induced resistin transactivation in gene reporter assays. These findings demonstrate that, in calcified AS, levels of Sirt1 are reduced whereas those of resistin are increased within aortic valve leaflets. Our results also suggest that this loss of Sirt1 expression alleviates its inhibition of resistin transcription in macrophages. Although the overall contribution of this process to the underlying mechanisms for AS disease development remains unresolved, these observations suggest that modification of Sirt1 expression and/or activity could represent a novel approach against inflammation in AS.

## Introduction

Calcific aortic valve disease is a progressive disorder that ranges from mild valve thickening without obstruction of blood flow, termed aortic sclerosis, to severe calcification and remodeling of the valve leading to impaired leaflet motion, or aortic stenosis (AS). Despite that calcific AS has become the most common cardiac disease in developed countries after hypertension and coronary artery disease [1], [2], [3], [4], the only permanent treatment remains valve replacement surgery, and no disease-modifying pharmacological tool is currently available. AS increases in prevalence with advancing age, afflicting 2–3% of the population by the age of 65 years [5].

Histopathologic and clinical data have suggested that AS is an active disease process akin to atherosclerosis with lipoprotein deposition and retention, chronic inflammation, recruitment of macrophages within the valvular tissue, and active leaflet calcification [6], [7], [8], [9]. These physiological modifications of the aortic valve are associated with changes in the expression of genes implicated in the adhesion and infiltration of blood cells, calcification, lipid retention, inflammation, and angiogenesis [10]. However, the cellular and molecular mechanisms implicated in the pathogenesis of this disease are still poorly understood [11].

Recently, higher circulating levels of the 12 kDa hormone resistin were associated with the severity of aortic valvular calcification and inflammation in older patients [12]. In humans, resistin is mostly secreted by macrophages [13]. In these cells, resistin promotes CD36 expression and lipid accumulation [14], [15], [16]. Likewise, reports linked resistin to atherosclerosis [17], [18], coronary artery calcification [18] and coronary artery disease [19].

Sirtuin 1 (Sirt1) is a class III deacetylase that targets histones and a variety of transcription factors and nuclear cofactors [20]. Sirt1 mediates many effects of caloric restriction in mammals [21]. In mice, Sirt1 decreases foam cell formation [22] and promotes eNOS-induced vasorelaxation [23]. Sirt1 was also demonstrated to be involved in the inhibition of inflammatory pathways in murine RAW264.7 macrophages by blunting the transcription factor activator protein 1 (AP-1) [24], [25] and reducing lipopolysaccharide (LPS)-induced tumor-necrosis factor (TNF)-α expression [25], [26]. In human U-937 macrophages, Sirt1 also downregulates the expression of metalloproteinase 9 (MMP9) [27], the activity of which being increased in aortic valve degeneration [28].

Since Sirt1-activating compounds have been shown to reduce macrophage inflammatory profiles *in vivo* [25] and to decrease resistin expression and secretion in adipocytes [29], [30], we hypothesized that a similar process could also take place in macrophages that infiltrate the aortic valve. Thus, we investigated the expression levels of Sirt1 in human stenotic aortic valves obtained from valve replacement surgery, and determined its direct effects on the transcription of resistin in macrophages.

## Methods

### Ethic Statement

Aortic valves were obtained from tissue bank at Institut Universitaire de Cardiologie et de Pneumologie de Québec (IUCPQ). IUCPQ ethic committee approved the study. Written informed consent was received from all participants.

### Tissue Collection

Stenosed valves were collected following valvular replacement surgery and control valves were obtained following heart transplant. Compared to control patients, those with AS were older (69 ± 2 vs 53 ± 3 years, p < 0.0001). There was no difference in body mass index, gender, smoking status, occurrence of vascular disease, dyslipidemia, and diabetes between groups. Frequency of hypertension was higher in AS patients (*p* = 0.01); accordingly, use of ACE inhibitors was also higher in this group (*p* = 0.001). Medication was otherwise similar between groups.

Valves were evaluated by a pathologist to confirm the presence of AS or the absence of clinical signs of AS and to assign a remodeling calcification score exactly as previously described [31]. Following their collection, valves were preserved either in RNA later (Ambion) or PFA 1% (Acros organics). One cusp of the valve was snap frozen in liquid nitrogen during the surgery. The frozen cusp was then crushed in liquid nitrogen and lysis buffer B was added (0,32 M sucrose, 10 mM TRIS, 1mM EDTA, 1mM β-mercaptoethanol, protease inhibitor (Roche)). Tubes were incubated 1 hour at 4°C with agitation then sonicated for a minute. Supernatant was collected and proteins were quantified.

### Immunoprecipitation of Sirt1 in Human Aortic Valves

40 µg of protein were precleared with protein A-sepharose beads (GE Healthcare, Baie d’Urfé, QC, Canada) for 2 h at 4°C and then centrifuged 5 min at 6000 g. Supernatants were immunoprecipitated using 2ug of anti-Sir2(Sirt1) antibody (Millipore) overnight at 4°C, and mouse IgG were used as negative control. Immunoprecipitates were washed once with IP buffer, twice with WB (0.25 M KCl in PBS) and then subjected to SDS–PAGE electrophoresis on polyacrylamide gels and electrophoretically transferred to polyvinylidene difluoride (PVDF) membranes. Membranes were incubated for 1 h at room temperature in blocking buffer [50 mM Tris–HCl pH 7.5, 150 mM NaCl, 0.02% Tween 20, 0.04% NP40 (wash buffer) and 5% dry milk powder], and overnight at 4°C with primary antibodies (anti-sirt1, Santa cruz sc-15404) in wash buffer containing 5% dry milk powder. PVDF membranes were then washed three times in wash buffer for 8 min and incubated for 1 h with anti-rabbit immunoglobulin G conjugated to horseradish peroxidase in wash buffer containing 5% dry milk powder at room temperature. After three washes for 8 min, the immunoreactive bands were detected by the chemiluminescent method. Quantification of density was computerized by multiplying pixels x intensity using Photoshop software.

### Immunohistochemistry

Sections of the valves were included in paraffin and were mounted on slides. Tissues sections were fixed in a 60% acetone and 40% methanol solution for 10 min at −20°C and left to dry for 5 minutes at room temperature then washed three times with TBS 1x. Immunohistochemistry was performed using EnVision+Dual link System-HRP (AEC) (Dako) following the manufacturer instructions. Briefly, residual peroxidase activity was quenched using Dual Endogenous Enzyme Block (Dako) for 10 minutes and tissues samples were incubated overnight at 4°C with an anti-Sirt1 antibody (SC-15404). Washed tissues sections were incubated with EnVision+Dual link System-HRP for 30 minutes and interactions were revealed by adding AEC substrate for 10 minutes. Sections were then counterstained with hematoxilin.

### Immunofluorescence

Tissue sections from stenotic and normal aortic valves were fixed in a 60% acetone and 40% methanol solution for 20 minutes at −20°C and washed in PBS. Sections were blocked with 1% BSA in PBS for 1 hour then incubated with primary antibodies against Sirt1 (sc-15404, Millipore) or no antibodies overnight at 4°C. Tissue sections were washed and incubated with secondary anti-rabbit FITC conjugated antibody (Santa-Cruz) and 1/1000 Hoechst (bisBenzimide H33258 (Sigma)) for 1 hour. Sections were washed and mounted using vectashield for observation.

### Cell culture and Treatments

U-937 and RAW264.7 monocytes (ATCC, Manassas, USA) were cultured in RPMI 1640 medium (GIBCO) prepared following the manufacturer’s instructions and supplemented with 10% fetal bovine serum (Sigma), 2mM glutamine (Sigma) and 1mM sodium pyruvate (GIBCO). Cells were plated at 200 000 cells/ml and induced to differentiate with 16 ng/ml phorbol 12-myristate 13-acetate (PMA; Fluka biochemika). Two days post-differentiation, cells were treated (for 24 hours) with growth medium supplemented with 1µg/ml lipopolysaccharide (Sigma) and 50 µM resveratrol (Sigma) as indicated in figure legends.

### Retroviral Infection

Viruses used in this study (pBabe or pBabe-Sirt1 for overexpression; pSuper, pSuper-Sirt1-scrambled or pSuper-Sirt1 RNAi for downregulation) were described previously [32]. Viruses were produced in 293T cells (ATCC, Manassas, USA) exactly as previously described [32]. Two days later, medium was harvested, filtered and transferred to RAW264.7 cells in the presence of polybrene (1µg/ml). Cells were selected using puromycin (2.5µg/ml) two days after the infection. Infected cells were grown using complete RPMI 1640 medium described above with 2.5µg/ml puromycin.

### RNA Extraction and Quantitative Real Time PCR

RNA was isolated from cultured cells by the phenol-chloroform method and from aortic valves using a RNeasy lipid tissue extraction kit (Qiagen). Reverse transcription of 1 µg of RNA was performed using qScript cDNA supermix (Quanta) following the manufacturer’s instructions. Quantitative real time PCR (qPCR) was performed using SYBR Green PCR Mastermix without MgCl_2_ (Invitrogen) using Rotor-Gene 3000 instrument (Corbett Research). Data were analyzed using the standard curve method and all genes of interest were normalized to the housekeeping gene L27 for human valves and Glyceraldehyde 3-phosphate dehydrogenase (GAPDH) for the cells. Primer sequences are: Human resistin: AGAGGCGCCTGCAGGATGAAAG / TGGAGGTGACGCTCTGGCAC; Human GAPDH: CGCCAGCCGAGCCACATC / GCCAGCATCGCCCCACTTGA; Human Sirt1: AGTTGCCACCCACACCTCTTCAT / GCCGCCTACTAATCTGCTCCTT; Mouse resistin: GCTGAGGGTCTGGAAATGAA / GGCCAGCCTGGACTATATGA; Mouse GAPDH: ACGGCAAATTCAACGGCACAGTCA / TGGGGGCATCGGCAGAAGG; Mouse Sirt1: CAGTGTCATGGTTCCTTTGC / CACCGAGGAACTACCTGAT.

### ELISA Assays

After incubation of cells with indicated treatment (see figure legend), fresh media was collected, centrifuged, and immediately processed with an ELISA kit (DY1359 R&D systems). Proteins were extracted from attached cells and quantified as described above. Data are presented as pg of secreted resistin per mL of media corrected for total protein content.

### Luciferase Assays

293T cells were transfected (120 000 cells per well in 24-well plates) using Fugene (Roche) for 12 h with 80 ng of c-jun, 150 ng of b-galactosidase and 150 ng of Sirt1 expression plasmids (Addgene), and co-transfected with 300 ng of a reporter plasmid containing 3.0 kb of the human resistin promoter. The next day, the transfection medium was removed and fresh medium was added with or without resveratrol (20 µM). Luciferase and b-galactosidase activities were measured 24 h later as described [33]. Results are shown as luciferase activity relative to that of b-galactosidase.

### Chromatin Immunoprecipitation

Cells were fixed in 1% formaldehyde followed by 2 washes with 1X PBS with 0.1% protease inhibitors. Nuclear pellets were obtained using chromatin immunoprecipitation (ChIP) lysis buffer (50 mM HEPES pH 7.5, 140 mM NaCl, Triton 1%) with 0.1% protease inhibitors. Sonication was carried out with Sonifier cell disrupter 185 (Branson) in ChIP lysis buffer. Immunoprecipitations were performed using 200 µl of sonicated cells and 5µg of antibody: anti-Sirt1 (sc-15404, Santa Cruz), anti-c-Jun (sc-45, Santa Cruz), or nonspecific rabbit IgG control (sc-2027; Santa Cruz). DNA was isolated using a PCR purification Kit (Qiagen). Controls were performed in each case with inputs from the original protein sample. The sequences of the primers used for the PCR are: AP-1 putative binding site (from −657 to – 648 bp upstream of resistin start codon): Forward 5′-CTGGAGTGCAGTGCTGTGAT-3′; Reverse 5′ AGACTGGTCAGGAGGTGGTG 3′; Non-specific binding: Forward 5′-AAGCAACCCAAGTGTCCATC-3′; Reverse 5′ CCATCTTGCTTTCTGCCTCT 3′.

### Statistical Analysis

Data are expressed as mean +/- S.E.M. Statistical significance was performed by ANOVA. A value of *p*< 0.05 was considered to denote statistical significance.

## Results

### Reduction in SIRT1 Levels in AS

**Figure 1:**
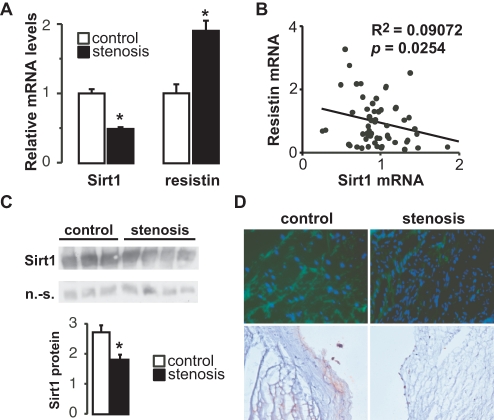
Low Sirt1 levels in aortic stenosis are inversely associated with resistin expression. **A**, Levels of Sirt1 and resistin mRNA levels in aortic valves from 11 control and 51 patients with AS. Gene mRNA was normalized to the housekeeping gene L27. **B**, Linear regression between relative resistin and Sirt1 mRNA levels. Each point represents a single patient. **C**, Sirt1 protein levels in aortic valves from control and AS patients. Bar graph depicts the signal intensity relative to that of a non-specific band. **D**, Sirt1 protein levels in aortic valve tissue from control and AS patients as visualized by immunofluorescence (IF, top panels) and immunohistochemistry (lower panels). In IF, FITC (green) signals were against Sirt1, whereas Hoescht (blue) signals strain nuclei. Representative pictures are shown. In all panels, * indicates a significant difference between groups.

To first test whether modulation Sirt1 could contribute to AS, gene expression levels were quantified in 11 control and 54 AS explanted valves. Messenger RNA concentrations of interleukin-1 beta (IL-1b, 0.35 ± 0.05 vs 1.47 ± 0.24, *p* = 0.04), and scavenger receptor A (SR-A, 0.56 ± 0.13 vs 1.62 ± 0.20, *p* = 0.02) were significantly increased in AS, whereas those of SR-B1 (*p* = 0.002) and peroxisome proliferator-activated receptor (PPAR)g (*p* = 0.006) were decreased (data not shown). Sirt1 mRNA levels were two-fold lower in AS samples than in controls (Fig. 1A, *p* = 0.01). As expected [12], resistin mRNA levels were two-fold increased in AS valves compared to those in control valves (Fig. 1A, *p* = 0.003). Interestingly, expression levels of resistin mRNA were inversely correlated with those of Sirt1 (*p* = 0.02) (Fig. 1B). Importantly, Sirt1 protein levels were also reduced in AS samples, as evidenced by western immunoblotting (Fig. 1C), immunohistochemistry (Fig. 1D) and immunofluorescence (Fig. 1D) assays. Taken together, these findings demonstrate that AS is associated with a robust reduction in Sirt1 expression, which was inversely correlated with that of resistin.

### Modulation of Resistin Expression by Sirt1 in Macrophages

**Figure 2:**
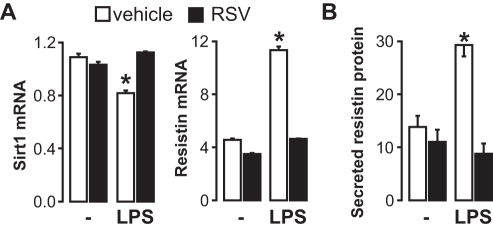
Activation of Sirt1 inhibits resistin expression in macrophages. **A**, Sirt1 and resistin mRNA levels in U937 macrophages treated for 24 h with LPS (1 µg/mL) and resveratrol (50 µM) or their respective vehicle. **B**, Secreted levels of resistin in U937 macrophages treated as in A. Bars are representative of 3 experiments done in triplicate. In all panels, * indicates a significant difference between groups.

**Figure 3:**
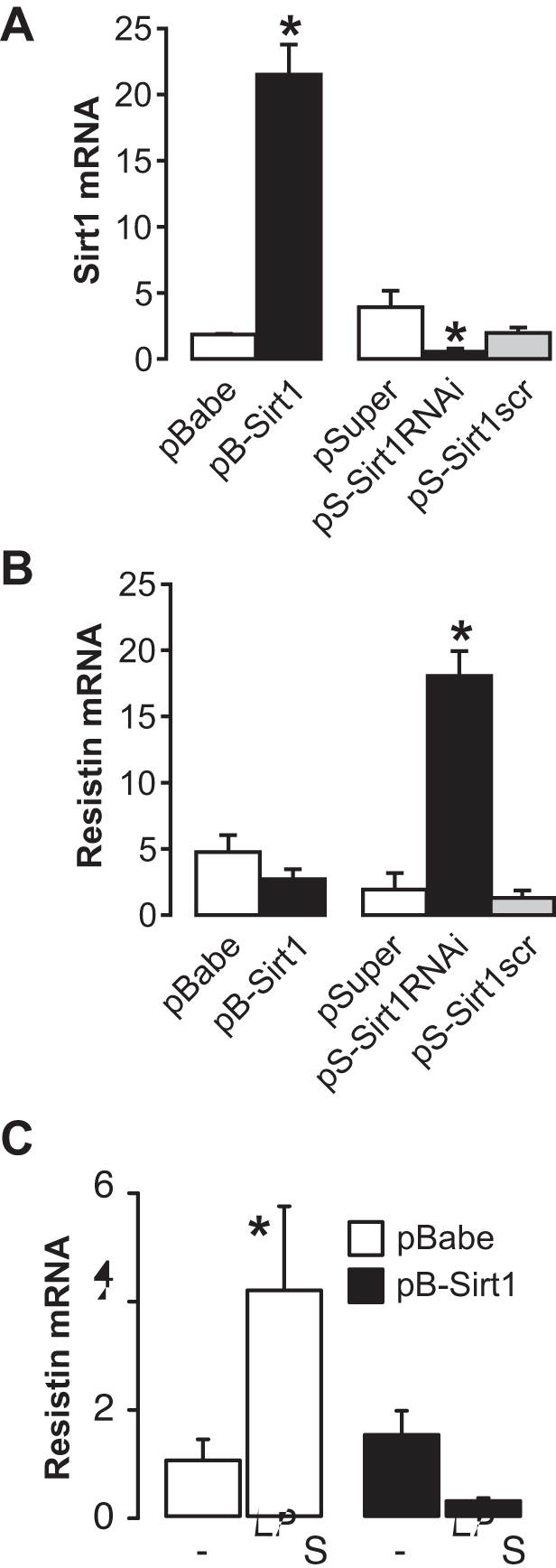
Downregulation of Sirt1 increases resistin expression in macrophages. Sirt1 (A) and resistin (B) mRNA levels in RAW264 macrophages infected with viruses for Sirt1 overexpression (pBabe-Sirt1) or downregulation (pSuper-Sirt1 RNAi) or their respective control vectors. Scr: scrambled sequence. (C) Resistin mRNA levels in pBabe- or pBabe-Sirt1-infected RAW264 macrophages treated for 24 h with or without LPS (1 µg/mL). Bars are representative of 3 experiments done in triplicate. In all panels, * indicates a significant difference between groups.

In humans, resistin is mostly expressed in macrophages, which infiltrate aortic valves upon the development of AS [28]. LPS strongly induces resistin expression *in vivo* and *in vitro* [34]. To determine whether Sirt1 directly controls resistin expression, human macrophages were incubated with LPS in the presence or absence of the Sirt1 activator resveratrol. LPS treatment diminished Sirt1 mRNA expression (*p* = 0.01) and increased resistin mRNA levels (*p* = 0.002) (Figure 2A). These changes were blunted by co-incubation with resveratrol (Fig. 2A), suggesting that activation of Sirt1 inhibits resistin expression. Importantly, activation of Sirt1 with resveratrol also impacted on the secreted levels of resistin protein (Fig. 2B). To further genetically assess the effect of Sirt1 on resistin expression, we infected macrophages with viruses for Sirt1 overexpression or downregulation (shRNA). In line with our findings in AS valves, downregulation of Sirt1 stimulated a 5-fold increase in resistin mRNA levels (Fig. 3B). Despite a ten-fold Sirt1 overexpression, basal resistin mRNA levels were not further reduced (Fig. 3B). However, overexpression of Sirt1 completely blunted the induction of resistin following LPS treatment (Fig. 3C). These results demonstrate that Sirt1 negatively regulates resistin expression in macrophages.

### Sirt1 Controls Resistin Expression via AP-1

**Figure 4:**
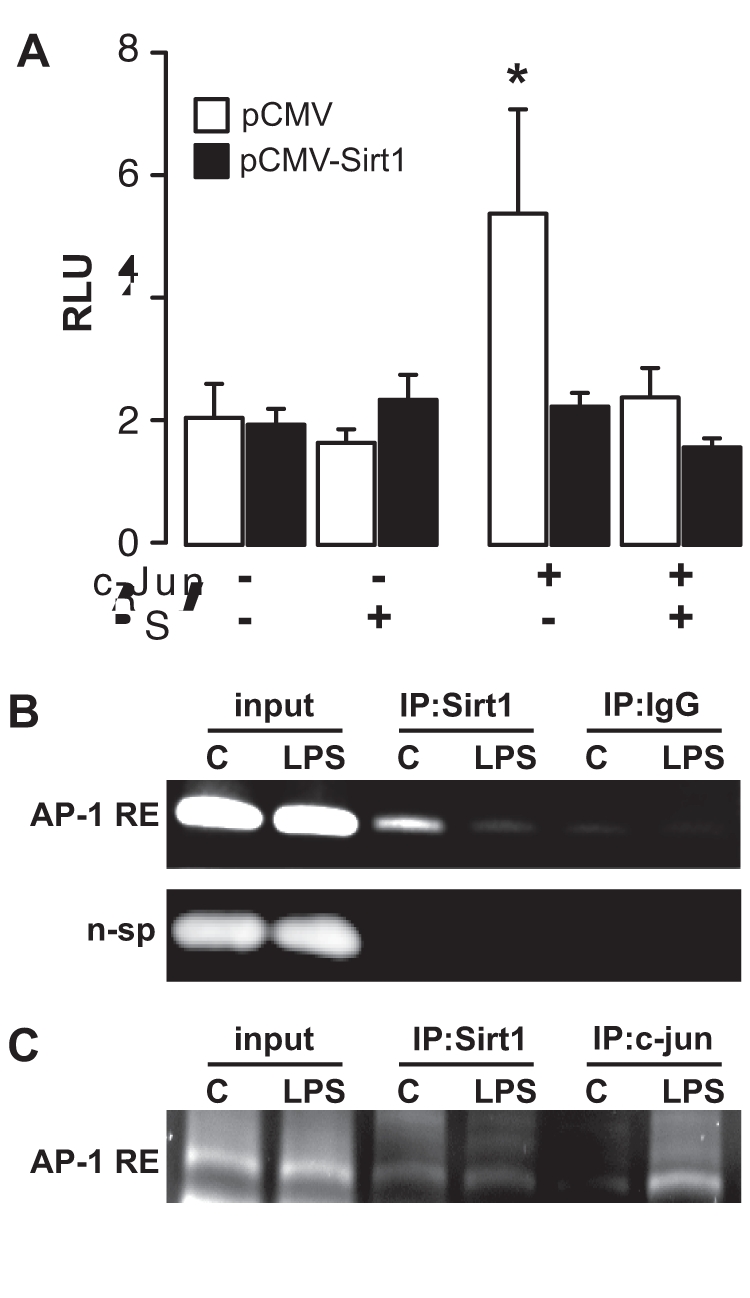
Repression of the human resistin promoter by Sirt1. **A**, Gene reporter assays using 3.0 kb of the human resistin promoter in 293 T cells transfected with c-Jun, Sirt1 or empty control plasmids and treated for 24 h with resveratrol (20 µM) or vehicle before quantification of the luminescent signal. Bars are representative of 3 experiments done in triplicate. * indicates a significant difference between groups. **B-C**, ChIP assay of the AP-1 response element on the resistin promoter using antibodies against Sirt1, IgG, or c-jun on U-937 cells treated with 1 µg/ml LPS or vehicle for 24 h.

The transcription factor AP-1 is a dimer formed by combination of members of the Jun, Fos, or ATF families, often including c-Jun. AP-1 controls the expression of basal and inducible genes, such as resistin [35], of which promoter regions contain the consensus sequence 5′-TGAG/CTCA-3′ also known as TPA responsive element (TRE) [36]. Since Sirt1 binds to and represses c-Jun transcriptional activity [37], we tested the possibility that Sirt1 inhibits resistin expression via a direct effect on its AP-1 responsive element. Consistent with a previous study using a synthetic AP-1-responsive promoter [37], gene reporter assays using plasmids containing 3.0 kb of the human resistin promoter showed that Sirt1 diminishes c-Jun-induced resistin expression (Fig. 4A). Consistent with Fig. 2, resveratrol treatment also reduced transactivation of the resistin promoter by c-Jun (Fig. 4A). Next, ChIP assays were performed on U-937 macrophages treated or not with LPS. Whereas Sirt1 was linked to an AP-1 response element on the resistin promoter in the basal state, this interaction was reduced in the presence of LPS (Fig. 4B). In line with this, binding of c-Jun to the same region was increased upon LPS treatment (Fig. 4C). Taken together, these results suggest that Sirt1 prevents c-Jun activity on the human resistin promoter, and that this process is relieved upon an inflammatory stimulus triggered by LPS.

## Discussion

It is becoming increasingly documented that the development of AS includes processes that have also been involved in atherosclerosis, such as a pro-inflammatory profile that promotes macrophage infiltration in aortic valves and tissue calcification. However, the molecular mechanisms regulating these events are not completely understood. In this study, we show that AS in humans is associated with low Sirt1 mRNA and protein expression levels. We further demonstrate that Sirt1 directly inhibits resistin promoter transactivation in cultured macrophages. Although this remains to be experimentally tested in the context of AS in vivo, we thus propose that the loss of Sirt1 could contribute to the development of AS-associated inflammation by allowing an upregulation of resistin expression in infiltrated macrophages.

Our results show that Sirt1 expression levels are lower in AS than in control valves. The extent of this reduction in Sirt1 was not associated with the severity of valve remodeling and calcification, as patients with mild cusp thickening but normal structural integrity had expression levels of Sirt1 similar to those with severe thickening, calcified nodules and fibrosa (data not shown). These findings suggest that the downregulation of Sirt1 occurs during the early stages of AS development. This decrease in Sirt1 levels might be related to the induction of AS-associated oxidative stress [38] driven by glucose intolerance and exposure to saturated fatty acids, as both were shown to reduce Sirt1 expression in human monocytes [39]. Because we were able to observe resistin expression in valves from control subjects who had no clinical signs of AS, it is likely that low-degree macrophage infiltration already took place. Thus, since levels of Sirt1 mRNA were affected in AS, the reduced transactivation of the Sirt1 promoter probably reflects a long-term change in the hormonal and cellular milieu of the infiltrated cells. Factors contributing to this reduction remain to be elucidated but could include the RNA-binding protein HuR, which has been shown to modulate Sirt1 mRNA stability upon oxidative stress [40].

Compared with control valves, calcified aortic valves displayed a pro-inflammatory profile including elevated levels of resistin and IL1b. The calcified valves also showed an mRNA expression profile facilitating foam cell formation. Levels of SR-A were increased and levels of PPARg and SR-B1 were decreased. Considering that SR-A is a mediator of lipid uptake [12] and that PPARg and SR-B1 are involved in lipid efflux [41], their modulation increases the amount of lipid present in the macrophages, stimulating the formation of foam cells as observed in mineralized valves [28].

In stenotic valves, there was a negative correlation between resistin and Sirt1 mRNA expression. To recreate the inflammatory condition associated with AS and hyperresistinemia [42], we treated macrophages with LPS, which was independently shown to increase resistin [14], [42], [43] and decrease Sirt1 expression [26] in these cells. In line with these results, modulation of Sirt1 by pharmacological and genetic tools demonstrated that Sirt1 directly impacts on resistin expression in macrophages. These findings are consistent with the negative effect of Sirt1 activation on resistin expression in 3T3-L1 cells [29], [30]. The physical interaction between Sirt1 and the c-jun component of AP-1 has recently been described to negatively regulate pro-inflammatory genes in macrophages [24], [25], [37]. Here, we show that Sirt1 binds to the resistin promoter and repress the transcriptional action of c-Jun in macrophages. Our data suggest that inflammation and oxidative stress implicated in the development of AS downregulates Sirt1 expression in infiltrated macrophages, which in turn could thus no longer repress AP-1, triggering resistin expression. Whereas independent studies have shown that the same interactions controls other pro-inflammatory genes such as TNFα in macrophages [25], it is possible that other regions on the resistin promoter are also involved in the repressive effect of Sirt1 [44], especially in the presence of AS-associated lower expression of PPARg [42], [45], [46].

In conclusion, our study indicates that Sirt1 levels are negatively associated with calcified AS in humans. In cultured macrophages, this loss allows an upregulation of resistin production. The regulation of resistin expression by Sirt1 is direct as Sirt1 binds the resistin promoter in the normal state. The decrease in Sirt1 levels in AS could allow an increase in c-jun transcriptional activity, triggering transcription of the resistin gene. Thus, modulation of Sirt1, along with that of LXR [47], TNFα [25] and MMP-9 [1], [27], [37] in macrophages, could represent novel avenues for the development of therapeutic solutions for AS, although this obviously remains to be tested in vivo. Interestingly, in two independent studies, levels of resistin were linked to the severity of calcification in vascular tissues [12], [18]. Moreover, a recent report showed that Sirt1 retards calcification of vascular smooth muscle cells in a rat model of renal failure [48]. Whether Sirt1 also impacts on AS calcification process per se is currently under investigation. Thus, the present study adds to the present intensive research on specific Sirt1 agonists for the treatment of cardiovascular diseases.
